# Molecular identification, antifungal susceptibility, and resistance mechanisms of pathogenic yeasts from the China antifungal resistance surveillance trial (CARST-fungi) study

**DOI:** 10.3389/fmicb.2022.1006375

**Published:** 2022-10-06

**Authors:** Qiqi Wang, Xuan Cai, Yun Li, Jianhong Zhao, Zhiyong Liu, Yan Jiang, Ling Meng, Yanming Li, Shiyang Pan, Xiaoman Ai, Fang Zhang, Ruoyu Li, Bo Zheng, Zhe Wan, Wei Liu

**Affiliations:** ^1^Department of Dermatology and Venereology, Peking University First Hospital, National Clinical Research Center for Skin and Immune Diseases, Research Center for Medical Mycology, Beijing Key Laboratory of Molecular Diagnosis on Dermatoses, Peking University, Beijing, China; ^2^Department of Clinical Laboratory, Renmin Hospital of Wuhan University, Wuhan, China; ^3^Institute of Clinical Pharmacology, Peking University First Hospital, Beijing, China; ^4^Department of Clinical Laboratory Medicine, Second Hospital of Hebei Medical University, Shijiazhuang, China; ^5^Department of Laboratory Medicine, Southwest Hospital, Army Medical University, Chongqing, China; ^6^Center for Clinical Laboratories, Affiliated Hospital of Guizhou Medical University, Guiyang, China; ^7^Lanzhou University Second Hospital, Lanzhou, China; ^8^Department of Clinical Laboratory, Xiangya Hospital, Central South University, Changsha, China; ^9^First Affiliated Hospital of Nanjing Medical University, Nanjing, China; ^10^Department of Medical Laboratory, Beijing Hospital, National Center of Gerontology, Institute of Geriatric Medicine, Chinese Academy of Medical Sciences, Beijing, China; ^11^Medical Research and Laboratory Diagnostic Center, Central Hospital Affiliated to Shandong First Medical University, Jinan, China

**Keywords:** pathogenic yeasts, invasive fungal diseases, *Candida* spp., antifungal susceptibility, triazoles, echinocandins, multidrug resistance

## Abstract

To have a comprehensive understanding of epidemiology and antifungal susceptibilities in pathogenic yeasts, the China Antifungal Resistance Surveillance Trial (CARST-fungi) study was conducted. All yeast isolates were identified by ribosomal DNA sequencing. Antifungal susceptibilities were performed using CLSI M27-A4 broth microdilution method. Sequence and expression level of resistant-related genes in resistant/non-wide-type (NWT) *Candida* isolates were analyzed. Totally 269 nonduplicate yeast isolates from 261 patients were collected. About half of the yeast isolates (127, 47.2%) were recovered from blood, followed by ascetic fluid (46, 17.1%). *C. albicans* remained the most prevalent (120, 44.6%), followed by *C. parapsilosis* complex (50, 18.6%), *C. tropicalis* (40, 14.9%), and *C. glabrata* (36, 13.4%). Fourteen (11.7%) *C. albicans* isolates and 1 (2.0%) *C. parapsilosis* isolate were resistant/NWT to triazoles. Only 42.5% (17/40) *C. tropicalis* were susceptible/WT to all the triazoles, with 19 (47.5%) isolates NWT to posaconazole and 8 (20%) cross-resistant to triazoles. Among *C. glabrata*, 20 (55.6%) and 8 (22.2%) isolates were resistant/NWT to voriconazole and posaconazole, respectively, and 4 (10.3%) isolates were cross-resistant to triazoles. Isavuconazole was the most active triazole against common *Candida* isolates. Except for 2 isolates of *C. glabrata* cross-resistant to echinocandins which were also NWT to POS and defined as multidrug-resistant, echinocandins exhibit good activity against common *Candida* species. All isolates were WT to AMB. For less common species, *Rhodotorula mucilaginosa* exhibited high MICs to echinocandins and FLC, and 1 isolate of *Trichosporon asahii* showed high MICs to all the antifungals except AMB. Among triazole-resistant *Candida* isolates, *ERG11* mutations were detected in 10/14 *C. albicans* and 6/23 *C. tropicalis*, while 21/23 *C. tropicalis* showed *MDR1* overexpression. Overexpression of *CDR1*, *CDR2*, and *SNQ2* exhibited in 14, 13, and 8 of 25 triazole-resistant *C. glabrata* isolates, with 5 isolates harboring *PDR1* mutations and 2 echinocandins-resistant isolates harboring S663P mutation in *FKS2*. Overall, the CARST-fungi study demonstrated that although *C. albicans* remain the most predominant species, non-*C. albicans* species accounted for a high proportion. Triazole-resistance is notable among *C. tropicalis* and *C. glabrata*. Multidrug-resistant isolates of *C. glabrata* and less common yeast have been emerging.

## Introduction

Invasive fungal diseases (IFDs) are life-threatening diseases with considerable morbidity and mortality, primarily occurring in immunocompromised and critically ill hosts (Bassetti et al., 2017). About 80% of IFDs were caused by *Candida* species, the third most frequently isolated microorganism of all infections in a worldwide ICU prevalence study (Kett et al., 2011). Although *C. albicans* continues to be the most prevalent *Candida* spp. causing IFDs, the past decades have witnessed changing epidemiology of IFDs shift to non-*albicans* spp., which may be more resistant to antifungal therapy (Bassetti et al., 2017). Other uncommon yeasts, such as *Cryptococcus* spp., *Saccharomyces* spp., *Trichosporon* spp., and *Rhodotorula* spp., although remained relatively rare in IFDs, were emerging as opportunistic pathogens and made diagnosis challenging and treatment suboptimal (Miceli et al., 2011). The management of IFDs was hindered owing to the paucity of rapid diagnostic assays (such as molecular identification methods). However, numerous clinicians still rely on traditional culture-based methods which are not rapid and sensitive. Therefore, empirical antifungal therapy often needs to be initiated and may contribute to increasing and inappropriate use of antifungals, which will not only alter *Candida* spp. distribution but also decrease antifungal susceptibility, making antifungal resistance an emerging problem worldwide (Lamoth et al., 2018).

There are 4 major classes of antifungals available to treat IFDs, including (1) polyenes, such as amphotericin B (AMB), destabilize membrane by binding to ergosterol; (2) triazoles, including fluconazole (FLC) itraconazole (ITC), voriconazole (VRC), posaconazole (POS), isavuconazole (ISA), target the enzyme 14-α-demethylase (Erg11p), a key step in the biosynthesis of ergosterol; (3) echinocandins, including caspofungin (CAS), anidulafungin (ANF), micafungin (MCF), block the catalytic subunit of the β-1,3 glucan synthase and thus inhibit cell wall biosynthesis; (4) pyrimidine analogs, such as 5-fluorocytosine (5-FC), are metabolized by fungal cells into fluorinated pyrimidines, which destabilize nucleic acids and result in growth arrest. Among these antifungals, triazoles represent the most widely prescribed antifungal class for IFDs. However, triazole-resistance has become a growing problem worldwide. The major mechanisms of triazole-resistance in *Candida* spp. include alteration in the *ERG11* gene encoding Erg11p, upregulation of the *ERG11* gene due to mutations in transcriptional factors such as *UPC2*, and overexpression of drug efflux pumps such as Mdr1p and Cdr1p/Cdr2p occurring mainly as a result of gain-of-function (GOF) mutations in transcription factors genes such as *MRR1*, *TAC1* and *PDR1* in *C. glabrata*. Echinocandins have now been recommended as first-line drugs for candidiasis (Pappas et al., 2016). Although the resistance rate remains generally low, echinocandin-resistance is rising as usage broadens among *Candida* spp., most notably *C. glabrata*, which was reported as high as >13% in some centers (Alexander et al., 2013). The mechanism of echinocandin resistance involves mutations in *FKS* genes encoding subunits of glucan synthase (Arastehfar et al., 2020). Different resistance mechanisms vary by species and geographic distribution, and the underlying mechanisms in some species have not been well defined. A better understanding of antifungal resistance mechanisms will provide insights to reclaim those antifungal classes as an option for empiric treatment of IFDs.

Under these circumstances, surveillance of IFDs plays a significant role in understanding epidemiology and antifungal susceptibility data to guide empirical therapy and aid antifungal stewardship efforts (Pfaller et al., 2019). The China Antifungal Resistance Surveillance Trial (CARST-fungi) study was a prospective national surveillance program for IFDs in mainland China. The study described the epidemiology and antifungal susceptibilities of clinical yeast isolates, including *Candida* spp., *Cryptococcus neoformans*, *Trichosporon asahii*, and other less common yeast species recovered from patients across major cities in China during 2019 and 2020. Additionally, to preliminarily elucidate the mechanisms underlying the resistant phenotypes, sequences of resistance-related genes *ERG11*, *PDR1*, *FKS*, and expression profiles of *ERG11* and efflux pump genes including *CDR1*, *CDR2*, *MDR1*, and *SNQ2*, were analyzed.

## Materials and methods

### Study design

The CARST-fungi study was a multi-center, prospective, observational, and laboratory-based study of IFDs with its inception in July 2019 and finished in June 2020. Nine “rank-A tertiary” hospitals distributed throughout 9 major cities in China took part in the study. All *Candida*, *Cryptococcus*, and other yeast isolates recovered from sterile sites including blood, other sterile body fluids (ascitic fluid, pleural fluid, cerebrospinal fluid [CSF]), pus, tissue from patients with invasive yeast diseases, bronchoalveolar lavage fluid (BALF), central venous catheter (CVC) tips, biliary tract fluid were collected. Additionally, yeast strains from considered colonizers such as urine, feces, sputum, and the genital tract were also included. For each episode of yeast isolation, the information including the patient’s age, gender, the ward location (e.g., emergency department, surgical, medical, and ICU), the time of sample collection, the specimen type, and the initial species identification made by the referring laboratory were collected. All isolates were sent to the Research Center for Medical Mycology at Peking University First Hospital, Beijing, China, for further study.

### Species identification

To ensure the accuracy of identification, all clinical yeast isolates were identified to the species level in the central laboratory. Six colonies from primary culture plates were subcultured and identified using sequence-based methods for the internal transcribed spacer (ITS) region, 28S ribosomal subunit (D1/D2), and the intergenic spacer (IGS, for *Trichosporon* spp. and *Cryptococcus* spp.), primers used for identification were listed in Supplementary Table S1. Those sequences were aligned using CBS database^1^ (Pfaller et al., 2012).

### Antifungal susceptibility testing

Antifungal susceptibility testing was performed according to the Clinical and Laboratory Standards Institute (CLSI) M27-A4 microbroth dilution method (CLSI, 2017), and the tested drugs including AMB (from North China Pharmaceutical Co. Ltd., Shijiazhuang, China), FLC, ITC, VRC, POS, ISA, CAS, ANF, MCF and 5-FC, (all from Harveybio Gene Technology Co. Ltd., Beijing, China) were prepared according to CLSI methods.

Minimum inhibitory concentrations (MICs) were determined after 24 h incubation at 35°C for *Candida* spp. and *Trichosporon* spp. and after 72 h incubation for *Cryptococcus* spp. MICs were read as the lowest drug concentration producing a prominent decrease in turbidity translating to 50% (triazoles, echinocandins, and 5-FC) or 100% (AMB) growth reduction compared with the drug-free control. Quality control was performed with each test run using *C. parapsilosis* ATCC22019 and *C. krusei* ATCC6258.

The interpretation of susceptibility was performed by applying the updated species-specific clinical breakpoints (CBPs) according to CLSI M60 document (CLSI, 2020a). In the absence of CBPs, isolates were defined as having a wild-type (WT) or a non-WT (NWT) drug susceptibility phenotype according to the epidemiological cutoff values (ECVs) as determined by the CLSI M59 document (CLSI, 2020b). Cross-resistance was defined as resistance to at least two antifungals of the same drug class. Multidrug-resistance was defined as resistance to at least two classes of antifungal drugs (Orasch et al., 2014).

### Resistance-related genes amplification and sequencing

Genomic DNA of resistant or non-WT yeast strains was extracted using the QIAamp DNA Mini Kit (Qiagen, Hilden, Germany). Primers to amplify the full open reading frame of *ERG11*, *PDR1* (for *C. glabrata*), and *FKS1*, *FKS2* for (*C. glabrata*) were designed (Supplementary Table S1) according to the genome of *C. albicans* SC5314, *C. glabrata* CBS138, *C. tropicalis* MYA-3404, and *C. parapsilosis* CDC317 from the Candida Genome Database^2^ as reference. The amplified products were sent to the BGI Company (Beijing, China) for sequencing and the sequences were aligned with the reference strain using the Clustal Omega.^3^

### RNA extraction and quantitative real-time reverse-transcription (RT)-PCR

Suspensions of *Candida* isolates cells (OD_600_, 0.1) freshly prepared in YPD medium were grown at 35°C to reach the mid-exponential phase (OD_600_, 0.6–0.8). The cells were washed twice with sterile water. Total RNA was extracted using the RNeasy Mini kit (QIAGEN Science, Maryland, USA) following the manufacturer’s instructions. The RNA was then treated with RNase-free DNase (Thermo Fisher Scientific, USA) according to the manufacturer’s recommendations. cDNA was synthesized using an Advantage RT-for-PCR kit (Clontech) according to the manufacturer’s instructions. RT-qPCR was performed on an Applied Biosystems ViiA7 Real-Time PCR system using SYBR green reagent (Applied Biosystems). Optimal thermal cycling conditions consisted of a 10-min initial denaturation at 95°C, followed by 40 cycles of denaturation at 95°C for 15 s, and annealing/extension at 60°C for 10 s. The experiments were carried out in triplicate for each data point. The cycle threshold (CT) value of the gene was normalized to that of internal control *ACT1* gene for *C. albicans*, *C. tropicalis*, *C. tropicalis*, and *RND5.8* gene for *C. glabrata* as ΔCT value. Relative gene expression (2^ΔΔCt^) was calculated as the fold change in expression of the isolates compared to the mean expression values in drug-susceptible control strains including *C. albicans* SC5314, *C. glabrata* CBS138, *C. tropicalis* ATCC01463, and *C. parapsilosis* ATCC22019. The primers used were listed in Supplementary Table S1.

### Statistical analysis

Comparisons were performed using SPSS software version 22 (SPSS, Chicago, IL, USA). Continuous variables were compared using the Mann–Whitney test, and categorical variables were analyzed using the χ^2^ test or Fisher’s exact test. A value of *p* <0.05 was considered statistically significant.

## Results

### Patient demographics

A total of 269 nonduplicate yeast isolates from 261 patients were collected, among which more than 1 isolate was recovered from 8 patients. Of the yeast isolates investigated, 161 (59.9%) were cultured from male patients, and 108 (40.1%) were from female patients. The patients’ ages ranged from 0 to 98 years (median, 57 years; interquartile range, 36 to 72 years). Most isolates (252/269, 93.7%) were from hospital inpatients (including those in ICUs [23.4%], medical wards [28.3%], surgical wards [33.8%], obstetrics and gynecology departments [5.2%], paediatrics departments [3.0%]), and remaining 6.3% were from patients in emergency settings.

### Distribution of species by specimen type

**Figure 1:**
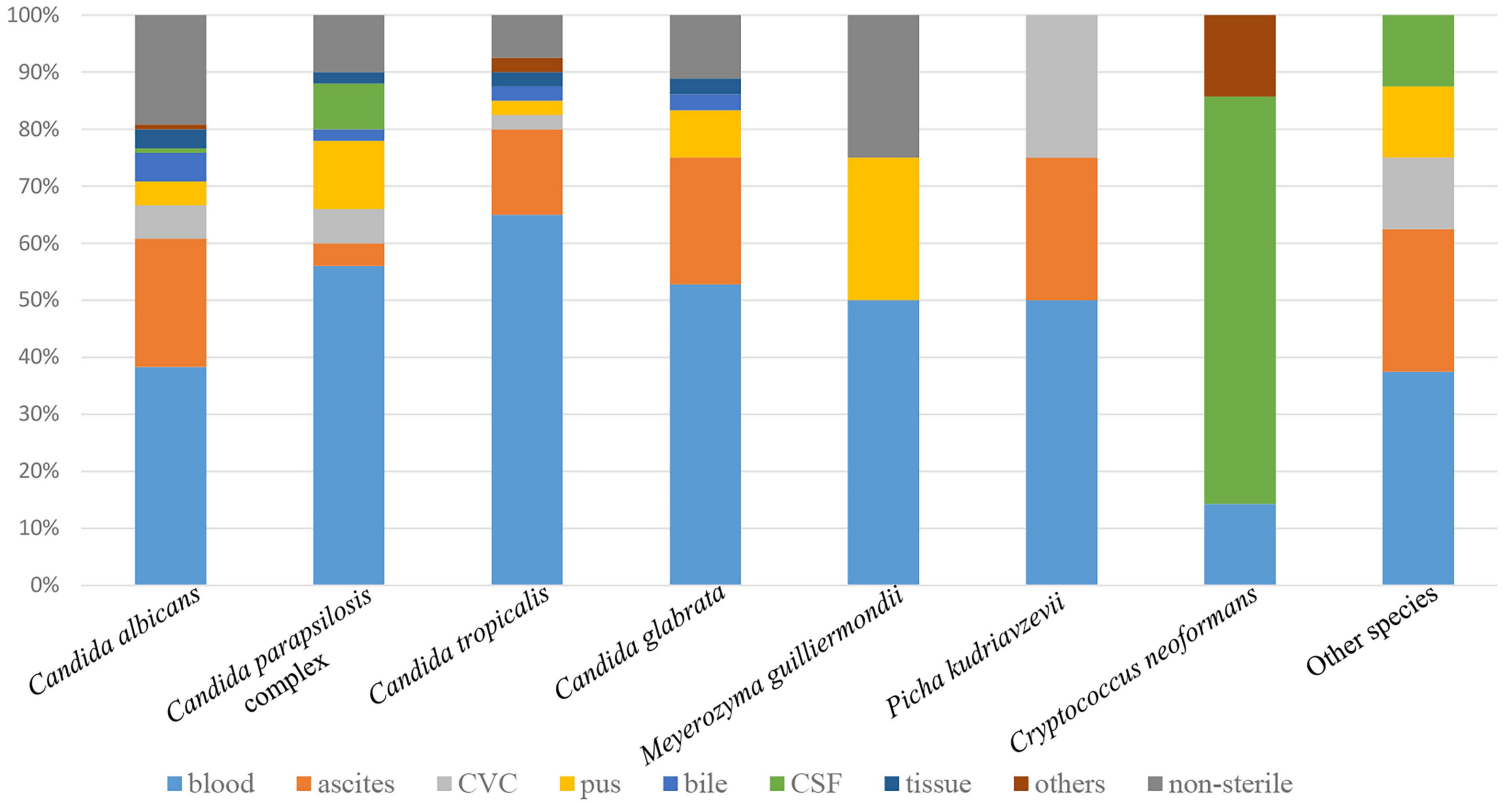
Distribution of yeast species by specimen type. Other species include 2 each of *Clavispora lusitaniae*, *Trichosporon asahii*, and *Wickerhamomyces anomalus*, 1 each of *Cyberlindnera fabianii* and *Rhodotorula mucilaginosa*. Other sterile sites include bone marrow and bronchoalveolar lavage fluid (BALF). Non-sterile samples include urine, feces, sputum, and genital tract secretion. CVC, central venous catheter; CSF, cerebrospinal fluid.

Of the various specimen types, about half of the yeast isolates (127, 47.2%) were recovered from blood, followed by ascitic fluid (46, 17.1%), pus (17, 6.3%), CVC (13, 4.8%), CSF (11, 4.1%), bile (9, 3.3%), tissue (7, 2.6%), and other sterile sites (3, 1.1%, including bone marrow, BALF; Figure 1). Thirty-six yeast isolates (13.4%) were from non-sterile samples and considered colonizers (such as urine, feces, sputum, and genital tract secretion). The proportion of non-*albicans Candida* isolates recovered from blood (78/127, 61.4%) was significantly higher than that recovered from other specimen types (58/142, 40.8%) (*p* < 0.01).

### Yeast species distribution

**Figure 2:**
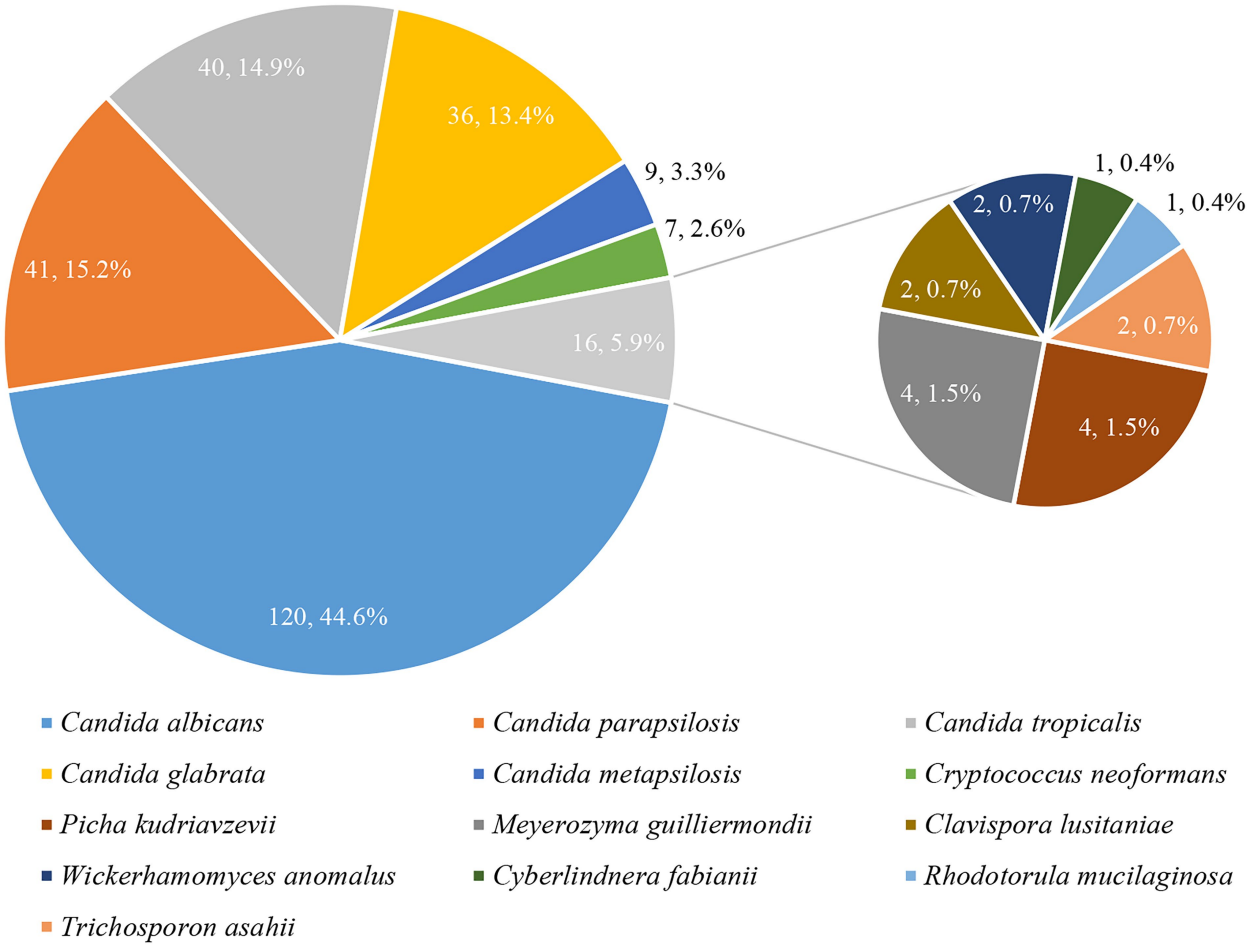
Species distribution of common and less common yeast isolates.

Thirteen species were identified among the 269 yeast isolates (Figure 2). *C. albicans* remained the most prevalent (120, 44.6%), followed by *C. parapsilosis* complex (including *C. parapsilosis sensu stricto* [41, 15.2%] and *C. metapsilosis* [9, 3.3%]), *C. tropicalis* (40, 14.9%), and *C. glabrata* (36, 13.4%). Other yeast species including 7 isolates of *Cryptococcus neoformans*, 4 each of *Picha kudriavzevii* (*Candida krusei*) and *Meyerozyma guilliermondii* (*Candida guilliermondii*), 2 each of *Clavispora lusitaniae* (*Candida lusitaniae*), *Trichosporon asahii*, and *Wickerhamomyces anomalus* (*Candida pelliculosa*), 1 each of *Cyberlindnera fabianii* (*Candida fabianii*) and *Rhodotorula mucilaginosa*, accounted for tiny proportion.

### Mixed species detection in the same specimen

Eight out of 261 samples (3.1%) in our study were co-isolated more than 1 yeast species (Table 1). Of the 8 specimens, 5 were from blood, 1 each from ascites, paracentesis fluid, and urine, among which 4 cases were *C. albicans*/*C. glabrata* mixed, 1 each *C. albicans*/*C. tropicalis mixed and C. parapsilosis sensu stricto*/*C. metapsilosis* mixed. Interestingly, 2 isolates of *T. asahii* in blood specimens were both co-isolated with *C. albicans*. Of note, most specimens contained yeast isolates that were resistant/NWT to at least one antifungal (Table 1).

**Table 1:** Distribution of co-isolated yeast species in specimens and their antifungal susceptibility profiles.

Specimen	Initial identification	Pathogens detected	Antifungal susceptibility MICs (μg/ml)									
			FLC	ITC	VRC	POS	ISA	AMB	ANF	MCF	CAS	5-FC
Blood	*C. parapsilosis*	*C. tropicalis*	4	0.25	0.5	0.5	0.25	1	0.12	0.06	0.06	>64
		*C. albicans*	0.5	0.06	0.008	0.03	0.008	1	0.06	0.008	0.06	<0.06
Blood	*C. glabrata*	*C. glabrata*	32	1	0.5	1	0.12	1	0.03	<0.008	0.03	<0.06
		*C. albicans*	0.12	0.06	0.008	0.015	0.015	0.5	0.12	0.008	0.12	0.06
Blood	*C. parapsilosis*	*C. parapsilosis*	0.25	0.12	0.008	0.06	0.008	0.5	1	1	0.5	0.06
		*C. metapsilosis*	2	0.25	0.03	0.12	0.008	0.5	0.5	0.5	0.25	0.06
Blood	*T. asahii*	*C. albicans*	4	0.25	0.5	0.06	4	0.5	0.015	0.015	0.015	0.5
		*T. asahii*	4	0.25	0.06	0.25	0.12	0.5	>8	>8	>8	4
Blood	*T. asahii*	*C. albicans*	4	0.25	1	0.12	0.008	1	0.06	0.008	0.03	64
		*T. asahii*	>256	>16	>8	>8	16	0.5	>8	>8	>8	>64
Paracentesis fluid	*C. albicans*	*C. albicans*	0.5	0.12	0.015	0.03	0.015	0.5	0.12	0.015	0.06	1
		*C. glabrata*	32	1	1	1	0.5	1	0.03	0.015	0.03	0.06
Urine	*C. glabrata*	*C. glabrata*	8	0.5	0.06	0.5	0.12	1	0.03	0.008	0.06	0.06
		*C. albicans*	4	0.06	0.06	1	0.015	1	0.06	0.008	0.06	0.06
Ascites	*C. glabrata*	*C. glabrata*	32	1	1	1	0.015	0.5	0.03	0.015	0.06	0.06
		*C. albicans*	0.5	0.12	0.015	0.03	0.008	0.5	0.12	0.008	0.06	0.06

FLC, fluconazole; ITC, itraconazole; VRC, voriconazole, POS, posaconazole; ISA, isavuconzaole; AMB, amphotericin B; ANF, anidulafungin; MCF, micafungin; CAS, caspofungin; 5-FC, 5-fluorocytosine; MICs, minimum inhibitory concentrations. MICs highlighted in red represent the isolate non-susceptible to the antifungal agent.

### *In vitro* susceptibility to triazoles

*In vitro* susceptibility of antifungal drugs against yeast species is shown in Table 2. Among the common *Candida* species, 5 (4.2%), 6 (5.0%), and 10 (8.4%) isolates of *C. albicans* were resistant/NWT to FLC, VRC, and POS, respectively. As for the *C. parapsilosis* complex, only 1 (2.4%) isolate of *C. parapsilosis sensu stricto* was cross-resistant to FLC and POS, while all the 9 *C. metapsilosis* isolates were WT to triazoles. However, only 42.5% (17/40) of *C. tropicalis* were susceptible/WT to all the triazoles, and 12 (30.0%), 3 (7.5%), 8 (20.0%), 19 (47.5%) isolates were resistant/NWT to FLC, ITC, VRC, and POS, respectively. Among *C. glabrata*, 2 (5.6%) isolates were resistant to FLC while the remaining 34 were susceptible-dose dependent (SDD), 20 (55.6%) and 8 (22.2%) isolates were resistant/NWT to VRC and POS, respectively, and all isolates were WT to ITC.

**Table 2:** Activities of 9 antifungal drugs against yeast species according to CLSI clinical breakpoints or ECVs.

Organism/Antifungal agents		FLC	ITC	VRC	POS	ISA	AMB	ANF	MCF	CAS	5-FC
*C. albicans*, *n* = 120	Breakpoints (*S*, *I*, *R*) (μg/ml)	*S* ≤ 2, SDD 4, *R* ≥ 8	/	*S* ≤ 0.1, *I* = 0.25–0.5, *R* ≥ 1	/	/	/	*S* ≤ 0.25, *I* = 0.5, *R* ≥ 1	*S* ≤ 0.25, *I* = 0.5, *R* ≥ 1	*S* ≤ 0.25, *I* = 0.5, *R* ≥ 1	/
	ECV (μg/ml)	/	/	/	0.06	/	2	/	/	/	/
	Range (μg/ml)	0.12–256	0.015–16	0.008–8	0.008–8	0.008–16	0.12–2	0.015–0.25	0.008–0.06	0.015–0.25	0.06–64
	MIC_50_ (μg/ml)	0.5	0.12	0.015	0.03	0.008	0.5	0.12	0.015	0.06	0.06
	MIC_90_ (μg/ml)	1	0.12	0.03	0.06	0.015	1	0.12	0.015	0.12	0.12
	CLSI S	111	/	109	/	/	/	120	120	120	/
	CLSI R	5	/	6	/	/	/	0	0	0	/
	WT	/	/	/	110	/	120	/	/	/	/
	non-WT	/	/	/	10	/	0	/	/	/	/
*C. parapsilosis* complex, *n* = 50											
*C. parapsilosis sensu stricto*, *n* = 41	Breakpoints (*S*, *I*, *R*) (μg/ml)	*S* ≤ 2, SDD 4, *R* ≥ 8	/	*S* ≤ 0.12, *I* = 0.25–0.5, *R* ≥ 1	/	/	/	*S* ≤ 2, *I* = 4, *R* ≥ 8	*S* ≤ 2, *I* = 4, *R* ≥ 8	*S* ≤ 2, *I* = 4, *R* ≥ 8	/
	ECV (μg/ml)	/	0.5	/	0.25	/	1	/	/	/	/
	Range (μg/ml)	0.25–8	0.03–0.5	0.008–0.03	0.03–0.5	0.008–0.25	0.25–1	0.06–2	0.015–2	0.12–1	0.06–0.25
	MIC_50_ (μg/ml)	0.5	0.12	0.015	0.03	0.008	0.5	2	1	0.5	0.06
	MIC_90_ (μg/ml)	1	0.12	0.03	0.06	0.015	1	2	2	1	0.12
	CLSI S	40	/	40	/	/	/	41	41	41	/
	CLSI R	1	/	0	/	/	/	0	0	0	/
	WT	/	41	/	40	/	41	/	/	/	/
	non-WT	/	0	/	1	/	0	/	/	/	/
*C. metapsilosis*, *n* = 9	Breakpoints (*S*, *I*, *R*) (μg/ml)	/	/	/	/	/	/	/	/	/	/
	ECV (μg/ml)	4	1	0.06	0.25	/	1	0.5	1	0.25	/
	Range (μg/ml)	0.25–2	0.03–0.25	0.008–0.06	0.03–0.25	0.008–0.06	0.5–1	0.12–0.5	0.015–0.5	0.06–0.25	0.06
	MIC_50_ (μg/ml)	1	0.12	0.03	0.06	0.008	0.5	0.25	0.5	0.12	0.06
	MIC_90_ (μg/ml)	2	0.25	0.06	0.25	0.015	1	0.5	0.5	0.25	0.06
	CLSI S	/	/	/	/	/	/	/	/	/	/
	CLSI R	/	/	/	/	/	/	/	/	/	/
	WT	9	9	9	9	/	9	9	9	9	/
	non-WT	0	0	0	0	/	0	0	0	0	/
*C. tropicalis*, *n* = 40	Breakpoints (*S*, *I*, *R*) (μg/ml)	*S* ≤ 2, SDD 4, *R* ≥ 8	/	*S* ≤ 0.12, *I* = 0.25–0.5, *R* ≥ 1	/	/	/	*S* ≤ 0.25, *I* = 0.5, *R* ≥ 1	*S* ≤ 0.25, *I* = 0.5, *R* ≥ 1	*S* ≤ 0.25, *I* = 0.5, *R* ≥ 1	/
	ECV (μg/ml)	/	0.5	/	0.12	/	2	/	/	/	/
	Range (μg/ml)	0.5–128	0.03–16	0.12–8	0.06–8	0.008–16	0.5–2	0.015–0.25	0.015–0.06	0.015–0.5	0.06–64
	MIC_50_ (μg/ml)	2	0.25	0.12	0.25	0.008	1	0.12	0.03	0.03	0.06
	MIC_90_ (μg/ml)	64	0.5	2	1	0.25	2	0.25	0.06	0.12	0.12
	CLSI S	18	/	27	/	/	/	38	40	39	/
	CLSI R	12	/	8	/	/	/	0	0	0	/
	WT	/	37	/	21	/	40	/	/	/	/
	non-WT	/	3	/	19	/	0	/	/	/	/
*C. glabrata*, *n* = 36	Breakpoints (*S*, *I*, *R*) (μg/ml)	SDD ≤ 32, *R* ≥ 64	/	/	/	/	/	*S* ≤ 0.12, *I* = 0.25, *R* ≥ 0.5	*S* ≤ 0.06, *I* = 0.12, *R* ≥ 0.25	*S* ≤ 0.12, *I* = 0.25, *R* ≥ 0.5	/
	ECV (μg/ml)	/	4	0.25	1	/	2	/	/	/	/
	Range (μg/ml)	4–128	0.5–2	0.06–4	0.25–4	0.008–2	0.25–1	0.015–4	0.008–8	0.03–16	0.06–0.25
	MIC_50_ (μg/ml)	32	0.5	0.5	1	0.12	1	0.03	0.015	0.06	0.06
	MIC_90_ (μg/ml)	32	1	1	2	0.5	1	0.12	0.015	0.12	0.06
	CLSI S	0	/	/	/	/	/	34	34	32	/
	CLSI R	2	/	/	/	/	/	2	2	2	/
	WT	/	36	16	28	/	36	/	/	/	/
	non-WT	/	0	20	8	/	0	/	/	/	/
*C. krusei*, *n* = 4	Breakpoints (*S*, *I*, *R*) (μg/ml)	/	/	*S* ≤ 0.5, *I* = 1, *R* ≥ 2	/	/	/	*S* ≤ 0.25, *I* = 0.5, *R* ≥ 1	*S* ≤ 0.25, *I* = 0.5, *R* ≥ 1	*S* ≤ 0.25, *I* = 0.5, *R* ≥ 1	/
	ECV (μg/ml)	/	1	/	0.5	/	2	/	/	/	/
	Range (μg/ml)	64	0.25–0.5	0.5	0.25–0.5	0.25–0.5	1–2	0.06–0.12	0.015–0.12	0.5	8–64
	MIC_50_ (μg/ml)	64	0.5	0.5	0.5	0.5	1	0.12	0.12	0.5	16
	MIC_90_ (μg/ml)	/	0.5	0.5	0.5	0.5	2	0.12	0.12	0.5	64
	CLSI S	/	/	4	/	/	/	4	4	0	/
	CLSI R	/	/	0	/	/	/	0	0	0	/
	WT	/	4	/	4	/	4	/	/	/	/
	non-WT	/	0	/	0	/	0	/	/	/	/
*Meyerozyma guilliermondii*, *n* = 4	Breakpoints (*S*, *I*, *R*) (μg/ml)	/	/	/	/	/	/	*S* ≤ 2, *I* = 4, *R* ≥ 8	*S* ≤ 2, *I* = 4, *R* ≥ 8	*S* ≤ 2, *I* = 4, *R* ≥ 8	/
	ECV (μg/ml)	8	2	/	0.5	/	2	/	/	/	/
	Range (μg/ml)	0.5–8	0.06–0.5	0.03–0.12	0.25–1	0.06–0.5	0.25–0.5	1–2	0.25–0.5	0.25–1	0.06
	MIC_50_ (μg/ml)	1	0.25	0.06	0.25	0.12	0.5	1	0.5	0.5	0.06
	MIC_90_ (μg/ml)	8	0.5	0.12	1	0.5	0.5	2	0.5	1	0.06
	CLSI S	/	/	/	/	/	/	4	4	0	/
	CLSI R	/	/	/	/	/	/	0	0	0	/
	WT	4	4	/	3	/	4	/	/	/	/
	non-WT	0	0	/	1	/	0	/	/	/	/
*Cryptococcus neoformans*, *n* = 7	Breakpoints (*S*, *I*, R) (μg/ml)	/	/	/	/	/	/	/	/	/	/
	ECV (μg/ml)	8	0.25	0.25	0.25	/	0.5	/	/	/	8
	Range (μg/ml)	1–8	0.03–0.25	0.015–0.12	0.015–0.25	0.008–0.12	0.25–0.5	>8	>8	>8	1–8
	M*I*C_50_ (μg/ml)	4	0.12	0.06	0.12	0.03	0.5	>8	>8	>8	4
	MIC_90_ (μg/ml)	8	0.25	0.12	0.25	0.12	0.5	>8	>8	>8	8
	CLSI S	/	/	/	/	/	/	/	/	/	/
	CLSI R	/	/	/	/	/	/	/	/	/	/
	WT	7	7	7	7	/	7	/	/	/	7
	non-WT	0	0	0	0	/	0	/	/	/	0
Uncommon yeast species with <3 isolates											
*Clavispora lusitaniae*, *n* = 2	MICs	1	0.25	0.015	0.06	0.015	0.5	1	0.12	0.5	0.06
		0.5	0.12	<0.008	0.06	0.008	0.5	0.25	0.12	0.25	<0.06
*Trichosporon asahii*, *n* = 2		>256	>16	>8	>8	16	0.5	>8	>8	>8	>64
		4	0.25	0.06	0.25	0.12	0.5	>8	>8	>8	4
*Wickerhamomyces anomalus*, *n* = 2		2	0.12	0.12	0.5	0.25	0.5	0.015	0.03	0.06	0.06
		8	0.25	0.25	0.5	0.25	0.5	0.015	0.015	0.06	0.06
*Cyberlindnera fabianii*, *n* = 1		1	0.25	0.03	0.5	0.03	0.25	0.03	0.03	0.03	0.06
*Rhodotorula mucilaginosa*, *n* = 1		>256	1	2	2	0.5	0.5	>8	>8	>8	0.06

FLC, fluconazole; ITC, itraconazole; VRC, voriconazole, POS, posaconazole; ISA, isavuconzaole; AMB, amphotericin B; ANF, anidulafungin; MCF, micafungin; CAS, caspofungin; 5-FC, 5-fluorocytosine; MICs, minimum inhibitory concentrations; ECVs, epidemiological cutoff values; WT, wide-type; non-WT, non-wild-type. *S*, susceptible; *R*, resistant; *I*, intermediate. MIC_50_/_90_ represent the MIC inhibiting the growth of 50 and 90% of the isolates, respectively.

For less common yeast species, all 4 isolates of *P. kudriavzevii* were susceptible/WT to other triazoles which were assumed to be intrinsically resistant to FLC; *M. guilliermondii* and *C. lusitaniae* were WT to triazoles except 1 isolate of *M. guilliermondii* was NWT to POS. Based on MIC_90_ since neither CBPs nor ECV values have been established for ISA and *Candida* spp., ISA was the most active triazole against *C. albicans* (MIC_90_, 0.015 μg/ml), *C. parapsilosis* (MIC_90_, 0.015 μg/ml), *C. tropicalis* (MIC_90_, 0.25 μg/ml), and *C. glabrata* (MIC_90_, 0.5 μg/ml), while comparable to other triazoles against *P. kudriavzevii*, *M. guilliermondii*, and other non-*Candida* spp. Two isolates of *W. anomalus* exhibited relatively less susceptible to FLC (MICs 2–8 μg/ml) and POS (MICs 0.5–1 μg/ml) than ITC, VRC, and ISA (MICs 0.12–0.25 μg/ml).

### *In vitro* susceptibility to echinocandins, AMB, and 5-FC

All isolates of *C. albicans* and *C. parapsilosis* were susceptible to echinocandins. Among *C. glabrata*, 2 isolates were cross-resistant to CAS, MCF, ANF (MICs 32 μg/ml, 8 μg/ml, 4 μg/ml, respectively) and 2 isolates were intermediate to CAS (MICs 0.25 μg/ml). There were 1 and 2 isolates of *C. tropicalis* exhibiting intermediate to CAS and ANF, respectively. Among the non-*Candida* yeasts, MICs of the echinocandins were consistently high for all isolates of *C. neoformans*, *R. mucilaginosa*, and *T. asahii* (all MICs >16 μg/ml) (Table 2).

All yeast isolates tested in this study were WT to AMB (MICs ≤2 μg/ml). No ECVs were defined for 5-FC against *Candida* spp., the MICs of 5-FC against 7 isolates of *C. albicans* and 2 isolates of *C. tropicalis* were > 64 μg/ml, while the MICs against the remaining *Candida* isolates were all ≤1 μg/ml. All isolates of *C. neoformans* were WT to 5-FC (ECV 8 μg/ml).

### Cross-resistance and multidrug-resistance

For yeast species with established CBPs or ECVs, 18 isolates were cross-resistant to at least 2 triazoles, including 5 (4.2%) isolates of *C. albicans*, 8 (20.0%) isolates of *C. tropicalis*, 4 (10.3%) isolates of *C. glabrata*, and 1 (2.4%) isolate of *C. parapsilosis sensu stricto*. Among triazole-resistant *Candida* isolates, 2 isolates of *C. albicans* and 1 isolate of *C. tropicalis* were also non-susceptible to 5-FC (MICs >64 μg/ml), and 2 isolates of *C. glabrata* were resistant to 3 echinocandins, which were defined as multidrug-resistance.

For less common species or those without CBPs or ECVs, 1 isolate of *R. mucilaginosa* exhibited high MICs to echinocandins and triazoles, and 1 isolate of *T. asahii* showed high MICs to all the antifungals tested except AMB (Table 2).

### Mutation and expression level of resistance-related genes in resistant/NWT isolates

From the sequencing results of *ERG11* in the 14 triazole-resistant/NWT *C. albicans* isolates, missense mutations were detected in 10 isolates, among which the most common substitution is Y132H exhibiting in 6 isolates. Four isolates without *ERG11* mutations exhibited overexpression of *ERG11*, *CDR1*, *CDR2*, or *MDR1* (Table 3). One isolate of *C. parapsilosis sensu stricto* cross-resistant to FLC and POS harbored R398I substitution in Erg11p as well as *CDR1* overexpression. Among the 23 triazole-resistant/NWT *C. tropicalis* isolates, only 6 isolates exhibited amino acid substitutions in Erg11p, among which S154F was detected in 1 isolate, Y132F with S154F was present in 5 isolates. Overexpression of *MDR1* was most frequent in triazole-resistant isolates (21/23), followed by *ERG11*, *CDR1*, and *CDR2*. In 25 triazole-resistant/NWT *C. glabrata* isolates, no mutations in *ERG11* despite of 5 isolates showed *ERG11* overexpression. Five isolates had *PDR1* modifications, among which P76S, P143T, D243N were detected in 3 isolates, 1 each harboring R250K and Y682C, respectively. Overall, there were 14, 13, and 8 isolates showed overexpression of *CDR1*, *CDR2*, and *SNQ2*, respectively. The entire sequences of *FKS1* and *FKS2* were determined in 2 echinocandin-resistant *C. glabrata* isolates, and S663P substitutions in *FKS2* were detected in both isolates (Table 3).

**Table 3:** Mutations and expression level of resistance-related genes in resistant/NWT *Candida* isolates.

Species/Isolates	Antifungal susceptibility/MICs^a^ (μg/ml)										Amino acid substitutions^b^			Gene expression^c^				
	FLC	ITC	VRC	POS	ISA	AMB	ANF	MCF	CAS	5-FC	*ERG11*	*PDR1*	*FKS2*	*ERG11*	*CDR1*	*CDR2*	*MDR1*	*SNQ2*
*Candida albicans*																		
10080	4	0.25	1	0.12	0.008	1	0.06	0.008	0.03	>64	WT	/	/	0.92	0.48	3.26^*^	0.77	/
10081	8	0.25	0.5	0.06	1	0.5	0.12	0.008	0.03	>64	T123I, Y132H	/	/	0.41	0.71	1.16	0.98	/
10108	>256	>8	>16	>8	>16	1	0.12	0.015	0.12	0.06	S263L, E266D	/	/	1.10	0.45	0.42	1.05	/
10568	1	0.25	0.03	0.12	0.008	1	0.12	0.008	0.06	0.06	S263L, E266D	/	/	2.03	2.03	1.50	2.04^*^	/
10570	>256	>8	>16	>8	>16	0.5	0.06	0.008	0.03	0.06	D116E, S263L, E266D	/	/	1.07	0.34	0.17	1.12	/
10573	1	0.12	0.03	0.12	0.03	1	0.12	0.015	0.12	0.25	D116E, K128T	/	/	0.42	2.19^*^	3.17	0.32	/
10574	2	0.25	0.5	0.12	0.5	0.5	0.12	0.015	0.06	0.25	D116E, K128T, Y132H, G465S	/	/	3.07^*^	0.25	0.25	0.82	/
10576	2	0.25	0.25	0.12	0.25	0.12	0.12	0.015	0.06	0.15	D116E, K128T, Y132H, G465S	/	/	0.25	0.46	0.30	3.03	/
10580	4	0.06	0.06	1	0.015	1	0.06	0.008	0.06	0.06	WT	/	/	0.65	0.57	7.07^***^	1.02	/
10648	256	>8	>16	>8	>16	0.5	0.06	0.015	0.06	<0.06	WT	/	/	7.54^**^	7.19^**^	0.98	1.93	/
10749	8	0.25	2	0.03	0.06	1	0.015	0.015	0.015	0.5	WT	/	/	2.19^*^	0.22	0.17	3.01^**^	/
10751	4	0.25	0.5	0.12	4	0.5	0.015	0.015	0.015	0.5	T123I, Y132H	/	/	1.02	2.13	0.78	2.31	/
10813	2	0.12	0.25	0.12	0.25	0.5	0.12	0.015	0.03	0.5	D116E, K128T, Y132H, G665S	/	/	1.14	0.26	0.25	2.02	/
10837	4	0.25	1	0.12	4	1	0.03	0.015	0.015	0.5	T123I, Y132H	/	/	0.82	0.41	2.81	0.59	/
*Candida parapsilosis*																		
10717	8	0.5	0.25	0.5	0.5	0.5	2	2	0.5	0.06	R398I	/	/	0.46	5.56^**^	/	0.64	/
*Candida tropicalis*																		
09956	>256	1	>16	2	0.5	2	0.12	0.03	0.06	0.06	Y132F, S154F	/	/	2.23	1.29	0.91	14.79^**^	/
10116	>256	1	>16	2	0.25	2	0.06	0.03	0.03	0.06	Y132F, S154F	/	/	6.62^*^	2.51	3.74	13.57^**^	/
10582	32	0.25	2	2	0.25	1	0.12	0.03	0.06	0.06	Y132F, S154F	/	/	1.83	0.92	0.97	4.76^**^	/
10641	64	0.25	2	1	0.25	1	0.12	0.06	0.03	<0.06	Y132F, S154F	/	/	10.28^**^	0.77	0.82	14.01^***^	/
09969	32	0.25	1	1	0.5	1	0.12	0.06	0.06	0.06	Y132F, S154F	/	/	1.70	2.45	9.07^**^	1.65	/
09973	128	0.25	4	1	0.12	1	0.12	0.03	0.03	0.06	WT	/	/	2.51	1.88	1.15	7.33^**^	/
10687	8	0.25	0.25	0.12	0.06	1	0.12	0.03	0.03	<0.06	WT	/	/	2.33	0.47	1.76	3.83^**^	/
10756	64	16	2	8	0.008	0.5	0.03	0.03	0.015	<0.06	WT	/	/	2.96	1.37	0.78	4.69^***^	/
10832	64	0.5	4	1	0.015	1	0.12	0.03	0.03	<0.06	S154F	/	/	4.37^***^	3.78^*^	6.74	13.26^**^	/
10041	4	0.5	0.25	0.5	0.06	1	0.5	0.12	0.06	0.06	WT	/	/	0.21	1.62	0.69	3.21^**^	/
10052	1	0.25	0.25	0.25	0.008	1	0.03	0.12	0.06	0.12	WT	/	/	1.52	1.34	2.35^*^	3.81^***^	/
10075	4	0.5	0.12	0.5	0.03	1	0.06	0.03	0.03	0.06	WT	/	/	4.55^*^	8.10	2.17	21.11^**^	/
10083	4	0.5	0.12	0.5	0.015	1	0.12	0.015	0.25	0.06	WT	/	/	2.03	1.34	1.16	6.42^***^	/
10117	4	0.5	0.12	0.25	0.03	1	0.12	0.03	0.25	0.06	WT	/	/	2.21	1.51	0.86	5.34^**^	/
10113	4	0.5	0.12	0.25	4	1	0.12	0.03	0.12	0.06	WT	/	/	1.90	3.01^*^	3.20^*^	7.37^***^	/
10120	256	0.03	0.06	0.12	0.5	1	0.06	0.03	0.03	0.06	WT	/	/	7.50^**^	5.90^***^	3.55^**^	28.11^**^	/
10629	4	0.5	0.12	0.25	0.03	0.5	0.06	0.03	0.03	0.06	WT	/	/	1.37	1.02	1.05	9.77^**^	/
10737	8	0.03	0.12	0.12	0.008	1	0.25	0.06	0.03	0.06	WT	/	/	4.85^**^	4.77^**^	2.36	15.49^**^	/
10752	8	0.5	0.06	0.06	0.008	1	0.03	0.03	0.015	0.06	WT	/	/	1.87	0.83	2.48	5.06^***^	/
10831	4	0.5	0.25	0.25	8	2	0.25	0.06	0.03	0.06	WT	/	/	2.74	0.76	0.66	7.77^***^	/
10739	4	0.5	0.5	0.25	0.015	2	0.12	0.03	0.03	0.06	WT	/	/	4.37^**^	3.78^*^	6.74^**^	13.26^***^	/
10639	4	0.25	0.25	0.25	0.25	1	0.12	0.06	0.06	>64	WT	/	/	3.77^**^	0.75	2.72	1.80	/
10642	4	0.5	0.12	0.25	0.008	1	0.03	0.03	0.03	0.06	WT	/	/	3.74^*^	11.59^***^	2.35	20.68^***^	/
*Candida glabrata*																		
10090	32	0.5	1	1	0.12	1	0.06	0.015	0.12	0.25	WT	WT	/	1.85	13.64^**^	19.97^**^	/	3.97
10123	128	>8	4	>8	2	1	0.12	0.015	0.12	0.06	WT	Y682C	/	0.54	2.64^*^	3.68^***^	/	1.33^*^
10124	32	1	1	1	0.12	1	0.06	0.008	0.12	0.06	WT	WT	/	5.33	10.27^*^	2.20	/	2.81
10578	16	1	2	2	0.015	1	0.03	0.008	0.06	0.06	WT	P76S, P143T, D243N	/	0.56	0.68	1.54	/	0.50
10045	16	>8	>16	>8	0.06	1	0.03	0.015	0.06	0.06	WT	WT	/	0.50	1.75	0.14	/	0.50
10055	32	1	1	2	0.12	0.5	0.03	0.008	0.03	0.06	WT	R250K	/	1.42^*^	7.22^***^	7.59^***^	/	2.92
10652	32	1	1	1	0.25	1	0.03	<0.008	0.03	<0.06	WT	WT	/	1.04	3.20^*^	1.10	/	0.14
09979	16	1	1	2	0.03	0.25	0.03	0.015	0.12	0.06	WT	WT	/	14.66^**^	6.56^***^	2.69	/	1.93
10742	64	1	2	2	0.015	0.5	0.06	0.015	0.12	<0.06	WT	WT	/	5.25^***^	1.45	3.11	/	1.98^*^
10720	4	2	0.12	4	0.25	1	4	8	>16	0.06	WT	P76S, P143T, D243N	S663P	4.35^***^	0.97	1.44	/	1.42
10722	4	2	0.12	4	0.25	1	4	8	>16	0.06	WT	P76S, P143T, D243N	S663P	1.12	3.01^**^	1.04	/	1.37
10082	32	1	1	1	0.5	0.5	0.03	0.015	0.06	0.06	WT	WT	/	1.84	0.59	4.51	/	1.64
10088	32	0.5	0.5	2	0.25	1	0.06	0.015	0.12	0.06	WT	WT	/	0.71	9.76	9.47	/	8.81^**^
10630	32	1	1	1	0.5	1	0.015	0.015	0.03	<0.06	WT	WT	/	2.27	4.15	13.36^***^	/	11.34^**^
10638	32	1	1	1	0.25	1	0.03	0.015	0.06	<0.06	WT	WT	/	4.96^**^	6.73^*^	0.74	/	1.40
10644	16	0.5	0.5	1	0.25	1	0.03	0.015	0.06	<0.06	WT	WT	/	0.33	6.72^**^	15.56^***^	/	4.24
10645	16	0.5	0.5	1	0.06	0.5	0.015	0.015	0.03	<0.06	WT	WT	/	0.67	10.62^***^	6.66^**^	/	2.89
10655	32	0.5	1	1	0.06	1	0.03	0.015	0.03	<0.06	WT	WT	/	2.61	0.36	1.15	/	0.99
10827	16	0.5	0.5	1	0.25	1	0.015	0.015	0.03	<0.06	WT	WT	/	0.13	21.96^**^	31.80^**^	/	2.74
10828	32	1	1	1	0.25	1	0.03	0.015	0.06	<0.06	WT	WT	/	0.16	2.47	14.10^***^	/	4.65
10829	32	1	1	1	0.06	1	0.03	0.015	0.06	<0.06	WT	WT	/	0.23	3.34	18.61^***^	/	3.98^*^
10830	32	1	1	1	0.015	1	0.03	0.015	0.03	0.06	WT	WT	/	7.50^*^	11.46^***^	3.74^**^	/	1.42
10838	16	0.5	0.5	1	0.25	1	0.03	0.015	0.06	<0.06	WT	WT	/	1.21	1.26	13.26^***^	/	7.38^**^
10718	32	0.5	1	1	0.015	1	0.06	0.015	0.06	0.06	WT	WT	/	3.44	3.50^*^	20.36^***^	/	4.68^*^
10728	32	0.5	0.25	2	0.25	1	0.12	0.015	0.12	0.06	WT	WT	/	0.65	4.54^**^	5.99^**^	/	7.85^*^

a Antifungal susceptibility MICs were determined by the CLSI M27-A4 broth microdilution method. FLC, fluconazole; ITC, itraconazole; VRC, voriconazole, POS, posaconazole; ISA, isavuconzaole; AMB, amphotericin B; ANF, anidulafungin; MCF, micafungin; CAS, caspofungin; 5-FC, 5-fluorocytosine. MICs highlighted in red represent the isolate non-susceptible to the antifungal agent.

b Sequences were align against that of *C. albicans* SC5314, *C. glabrata* CBS138, *C. tropicalis* MYA-3404, and *C. parapsilosis* CDC317 from the Candida Genome Database (http://www.candidagenome.org/) as reference.

c Quantification was performed using real-time PCR. Values are averages from three independent experiments and relative gene expression (2^ΔΔCt^) was calculated as the fold change in expression of the isolates compared to the mean expression values in drug-susceptible control strains including *C. albicans* SC5314, *C. glabrata* CBS138, *C. tropicalis* ATCC01463, and *C. parapsilosis* ATCC22019. Statistical significant overexpression genes relative to that of control strains were indicated as *p < 0.05; **p < 0.01; ****p < 0.0001.

## Discussion

To our knowledge, there are several excellent surveillance studies of IFDs worldwide, such as the SENTRY Antimicrobial Surveillance Program (Pfaller et al., 2019), the ARTEMIS DISK study (Pfaller et al., 2010), the SCOPE Program (Wisplinghoff et al., 2014), and so on. In China, the national-wide surveillance studies include the China Hospital Invasive Fungal Surveillance Net (CHIF-NET) study, which has provided useful data on the epidemiology of IFDs in mainland China (Xiao et al., 2018b), and China-SCAN study determining species distribution and antifungal susceptibility of invasive *Candida* infection (ICI) in ICU across China (Liu et al., 2014). The CARST-fungi study is another multi-center surveillance study of IFDs in mainland China with its inception in July 2019. Totally 9 hospitals participated during the first year with the collection of 269 yeast isolates. As expected, the four major *Candia* species, *C. albicans*, *C. parapsilosis* complex, *C. tropicalis*, and *C. glabrata*, accounted for predominate proportion. Although *C. albicans* remained the most prevalent (120, 44.6%), the proportion of non-*albicans Candida* species was over 50%, especially have been the primary causative pathogen of candidemia, in accordance with previous studies from China (Xiao et al., 2018a; Song et al., 2020), Asia-Pacific regions, and European countries (Pappas et al., 2018).

Mixed species detection in the same specimen was another significant result of our study. Totally, 8 out of 261 samples (3.1%) in our study were co-isolated with more than 1 yeast species, among which 4 cases were *C. albicans*/*C. glabrata* mixed, 1 each *C. albicans*/*C. tropicalis mixed and C. parapsilosis sensu stricto*/*C. metapsilosis* mixed, and 2 cases of *C. albicans* /*T. asahii* co-isolated. Indeed, mixed yeast infections have been detected in 8.78% of the culture-positive samples in an extensive study of 6,192 clinical yeast isolates (Cassagne et al., 2016). Similarly, an 18-year report from a tertiary-care university hospital revealed the incidence of mixed fungaemia was 3.7% (33/883) (Gulmez et al., 2020). Most mixed species in our study were *C. albicans*/*C. glabrata*, in accordance with the above-mentioned studies. Several studies revealed that *C. albicans* and *C. glabrata* are frequently co-isolated and co-adhesion *in vitro* (Tati et al., 2016), demonstrating enhanced invasion and increased tissue damage (Silva et al., 2011; Alves et al., 2014), which probably can be explained for the high proportion of the co-culture of these two species. Of note, most specimens contained yeast isolates that were non-susceptible to one or more antifungals. Mixed infections may further bring additional issues for treatment, especially infected by more than one fungal species with different drug susceptibilities, which highlights that mycological analysis of clinical samples should also reliably detect mixed fungal species, especially those involving yeasts species with a particular antifungal resistance profile. A previous study suggested that detection of mixed infection increased significantly after subculture from yeast-positive blood-culture bottles and routine use of chromogenic agar (Gulmez et al., 2020).

First-line drugs treating IFDs, including echinocandins and triazoles, are relatively effective, but the emergence of antifungal resistance is a matter of concern and poses a global threat (Pappas et al., 2018). In our study, most *C. albicans* and *C. parapsilosis* complex are susceptible to all the antifungals tested. Only 1 isolate (2.0%) of *C. parapsilosis sensu stricto* exhibited triazole-resistance, similar to the global (Pfaller et al., 2019) and domestic data (Wang et al., 2016; Xiao et al., 2018a; Song et al., 2020). The overall triazole-resistant rate of *C. albicans* was 11.6% (14/120), higher than the global data (<1%) (Pfaller et al., 2019). However, triazole susceptibility of *C. tropicalis* and *C. glabrata* was low, with only 42.5% (17/40) *C. tropicalis* and 30.5% (11/36) *C. glabrata* susceptible/WT to all tested triazoles. Of particular note, the POS-resistant rate of *C. tropicalis* isolates was 47.5% and the VRC-resistant rate of *C. glabrata* isolates was 55.6%, consistent with previous reports (Xiao et al., 2018a; Song et al., 2020) and much higher than that of past decade (Liu et al., 2014).

Another noteworthy finding of our study was the emergence of echinocandin resistance, although exhibiting excellent activity to most common *Candida* isolates. Two isolates of *C. glabrata* were cross-resistant to all the echinocandins tested, which also NWT to POS and defined as multidrug-resistant. Echinocandin-resistance among *C. glabrata* isolates ranges from 3–5% in population-based studies, and some centers even report as high as 10–15% (Farmakiotis et al., 2014), but it is less than 1% in China (Hou et al., 2017). Alarmingly, *C. glabrata* often presents as multidrug-resistance, with nearly one-third of echinocandin-resistant isolates also being non-susceptible to triazoles, leaving extremely few options to treat patients infected with multidrug-resistant isolates (Healey and Perlin, 2018). Emerging of multidrug-resistance is a significant threat to the treatment of IFD and urges our research group to explore the resistance mechanism of the *C. glabrata* isolates, demonstrating mutations in *FKS2* explaining echinocandin-resistance and overexpression of *CDR1* and *ERG11* contributing to triazole-resistance (Wang et al., 2021).

Except for the above-mentioned common *Candida* species, less common and cryptic yeast species were also identified in this study, among which *C. metapsilosis* accounted for a high proportion of *C. parapsilosis* complex (9/50), indicating the proportion was underestimated. Interestingly, *C. metapsilosis* and *C. parapsilosis* were co-cultured, highlight the significance of subculture and accurate identification. One isolate of *C. fabianii* was identified in our study. *C. fabianii* infections have rarely been reported (Park et al., 2019) and only one case has been reported in China which causes blood infection in a premature infant (Wu et al., 2013). Additionally, *W. anomalus* (previously named *C. pelliculosa*) accounted for a low rate (<1%) and was similar to previous national surveillance in China (Liu et al., 2014), and to our knowledge, one of which in our study is the first isolate of *W. anomalus* reported to be cultured from CSF in China. *W. anomalus* isolates in our study were less susceptible to FLC (MICs 2–8 μg/ml) and POS (MIC, 0.5 μg/ml), while AMB, echinocandins, and 5-FC showed good *in vitro* activity, but recent research revealed that *W. anomalus* isolates showed high MICs against all triazoles tested and 5-FC (Zhang et al., 2021). As for other yeast genera, *C. neoformans*, *R. mucilaginosa*, and *T. asahii* were included in our study. As expected, most *C. neoformans* isolates were isolated from CSF, which is believed to be the prevalent pathogen of fungal meningitis. *R. mucilaginosa* is a multidrug-resistant pathogen with the ability to cause nosocomial infection (Huang et al., 2022), which exhibited high MICs to all the triazoles and echinocandins. Recently, *Trichosporon* spp. which is usually associated with superficial mycosis has recently been recognized as an emergent fungal pathogen capable of causing invasive infections (Padovan et al., 2019). Besides intrinsic echinocandins-resistance, one isolate of *T. asahii* in our study showed high MICs to all triazoles and 5-FC. Several studies have estimated antifungal profiles of rare yeast species (Stavrou et al., 2020), demonstrated that high MICs against azole drugs and echinocandins were common and resistance rates in yeast species are dynamic and variable between medical institutions and countries (Desnos-Ollivier et al., 2021), highlighting the need for an accurate species identification of yeast isolates, which is essential for proper management of patients and prevention of emergence of drug resistance.

Finally, sequence of *ERG11* genes (and *PDR1* in *C. glabrata*) and expression level of *ERG11* and efflux pump genes in triazole-resistant *Candida* isolates and sequence of *FKS* genes in echinocandin-resistant isolates were determined. *ERG11* mutations were found in 10 of 14 triazole-resistant *C. albicans* isolates and 6 of 23 *C. tropicalis* isolates, the most common substitution is Y132H in *C. albicans,* and Y132F with S154F in *C. tropicalis*, similar to Y132F and S154F were the most common substitutions in Erg11p of *C. tropicalis* and proven to mediate triazole resistance (Jiang et al., 2012; Castanheira et al., 2020). Except for mutations of drug target, overexpression of *MDR1* seems as the predominant mechanism of *C. tropicalis* triazole-resistance in our study. Despite of no *ERG11* mutation was detected in triazole-resistant *C. glabrata* isolates, 5 isolates exhibited *ERG11* overexpression, which was considered to be less common in *C. glabrata*. *PDR1* modifications were detected in 5 isolates, with 3 isolates harboring P76S, P143T, D243N and 1 each harboring R250K and Y682C, respectively. The latter two isolates showed high expression level of ABC transporter genes including *CDR1*, *CDR2*, and *SNQ2*, indicating those *PDR1* modifications maybe GOF mutations resulting in up-regulation of downstream ABC transporter genes, which need further verification. The entire sequences of *FKS1* and *FKS2* were determined in 2 echinocandin-resistant *C. glabrata* isolates and S663P substitutions in *FKS2* were detected in both isolates. Notably, there are numerous isolates without modifications in well-known drug targets or up-regulation of transporter genes, the resistant mechanisms remain to be elucidated.

There are some limitations in our study. First, this paper concluded the first-year data of the CARST-fungi study, which encompass only nine hospitals cannot reveal the dynamic change year by year. The study will continue and more centers will join in to enhance the comprehension of epidemiology and antifungal susceptibilities in pathogenic yeasts across China. Second, the information of the patients is incomplete including mycoses, antibiotics used, basal conditions and other more detailed clinical characteristics. These data need to be included in future study. Nevertheless, this study provides important epidemiological findings of species distribution, antifungal susceptibility profile, and preliminary resistant mechanism exploration, which are instrumental in designing strategies for better management of yeast infections in China.

In conclusion, the CARST-fungi study demonstrated that although *C. albicans* remain the most predominant species, non-*C. albicans* species accounted for a high proportion, especially as the causative pathogen of fungemia. Triazole-resistance is notable among *C. tropicalis* and *C. glabrata* and multidrug-resistant isolates of *C. glabrata* and less common yeast have been emerging.

## Data availability statement

The original contributions presented in the study are included in the article/Supplementary material, further inquiries can be directed to the corresponding author.

## Author contributions

WL designed the experiments, supervised the data analysis, and contributed to funding acquisition. WL, YL, BZ, and RL administered the project. XC, JZ, ZL, YJ, LM, YML, SP, XA, and FZ collected all the isolates and provided clinical information. QW contributed to the species identification, antifungal susceptibility testing, molecular biology experiments, and original draft preparation. YL and ZW supervised the transformation, collection, and storage of the isolates. QW, XC, and WL contributed to writing and reviewing the manuscript. All authors contributed to the article and approved the submitted version.

## Funding

This study was supported by the National Key Research and Development Program of China (2021YFC2302005) and National Natural Science Foundation of China (81971912).

## Conflict of interest

The authors declare that the research was conducted in the absence of any commercial or financial relationships that could be construed as a potential conflict of interest.

## Publisher’s note

All claims expressed in this article are solely those of the authors and do not necessarily represent those of their affiliated organizations, or those of the publisher, the editors and the reviewers. Any product that may be evaluated in this article, or claim that may be made by its manufacturer, is not guaranteed or endorsed by the publisher.

## Footnotes

^1^ http://www.cbs.knaw.nl/

^2^ http://www.candidagenome.org/

^3^ https://www.ebi.ac.uk/Tools/msa/clustalo/
