# Supplementary material for: Molecular identification, antifungal susceptibility, and resistance mechanisms of pathogenic yeasts from the China antifungal resistance surveillance trial (CARST-fungi) study
Source: Front Microbiol. 2022 Oct 6;13:1006375. doi: 10.3389/fmicb.2022.1006375 (PMC9583154; doi:10.3389/fmicb.2022.1006375)
Supplement: Supplementary file 1 [file Table_1.DOCX]

Supplementary Material

**Table S1. List of primers used for PCR amplification and sequencing of target genes.**

| **Primer Name** | **Sequence**  **(5′-3′)** | **Target gene / Purpose** |
| --- | --- | --- |
| ITS1 | TCCGTAGGTGAACCTGCGG | ITS /PCR and sequencing |
| ITS4 | TCCTCCGCTTATTGATATGC |  |
| NL1 | GCATATCAATAAGCGGAGGAAAAG | D1/D2 /PCR and sequencing |
| NL4 | GGTCCGTGTTTCAAGACGG |  |
| IGSF | ATCCTTTGCAGACGACTTGA | IGS /PCR and sequencing |
| IGSR | GTGATCAGTGCATTGCATGA |  |
| Ca-erg11F | ATGGCTATTGTTGAAACTGTCATTGA | Ca*ERG11* /PCR and sequencing |
| Ca-erg11R | TTAAAACATACAAGTTTCTCTTTTTT |  |
| Ca-ACT1a | TTGGTGATGAAGCCCAATCC | Internal control of Ca real-time PCR |
| Ca-ACT1b | CATATCGTCCCAGTTGGAAACA |  |
| Ca-erg11a | TTTAGTTTCTCCAGGTTATGCTCAT | real-time PCR for Ca*ERG11* |
| Ca-erg11b | ATTAGCTTTGGCAGCAGCAGTA |  |
| Ca-CDR1a | ATTCTAAGATGTCGTCGCAAGATG | real-time PCR for Ca*CDR1* |
| Ca-CDR1b | AGTTCTGGCTAAATTCTGAATGTTTTC |  |
| Ca-CDR2a | ATCTACTCCTGGAAGCAC | real-time PCR for Ca*CDR2* |
| Ca-CDR2b | GCATAGCACCTTTATTGA |  |
| Ca-MDR1a | GAGTTAAACATTTCACCCTC | real-time PCR for Ca*MDR1* |
| Ca-MDR1b | AACAAGATACCACCGACA |  |
| Cg-erg11F | ATGTCCACTGAAAACACTTC | Cg*ERG11* /PCR and sequencing |
| Cg-erg11R | CTAGTACTTTTGTTCTGGATGTC |  |
| RND5.8a | CTTGGTTCTCGCATCGATGA | Internal control of Cg real-time PCR |
| RND5.8b | GGCGCAATGTGCGTTCA |  |
| Cg-erg11a | ATTGGTGTCTTGATGGGTGGTC | real-time PCR for Cg*ERG11* |
| Cg-erg11b | TCTTCTTGGACATCTGGTCTTTCA |  |
| Cg-CDR1a | TAGCACATCAACTACACGAACGT | real-time PCR for Cg*CDR1* |
| Cg-CDR1b | AGAGTGAACATTAAGGATGCCATG |  |
| Cg-CDR2a | GTGCTTTATGAAGGCTACCAGATT | real-time PCR for Cg*CDR2* |
| Cg-CDR2b | TCTTAGGACAGAAGTAACCCATCT |  |
| Cg-SNQ2a | ACCATGTGTTCTGAATCAATCAAT | real-time PCR for Cg*SNQ2* |
| Cg-SNQ2b | TCGACATCATTACAATACCAGAAA |  |
| Ct-erg11F | ATGGCTATTGTTGATACTGCC | Ct*ERG11* /PCR and sequencing |
| Ct-erg11R | CTAAACCATACAAGTATCTC |  |
| Ct-ACT1a | CTCTTCTCAATCATCTGCTA | Internal control of Ct real-time PCR |
| Ct-ACT1b | TCTAACATCCAAGTCACATT |  |
| Ct-erg11a | TTGATTGATTCCTTGTTGGTTA | real-time PCR for Ct*ERG11* |
| Ct-erg11b | CATCTTGTAATTGTGGTTGTTC |  |
| Ct-CDR1a | AGACAATCAGAGCACACT | real-time PCR for Ct*CDR1* |
| Ct-CDR1b | AACCGAAGACAATATCAATCC |  |
| Ct-CDR2a | TGGCCAAGTAACCTCAGAGC | real-time PCR for Ct*CDR2* |
| Ct-CDR2b | ACACCCCGACCAGTAACAGT |  |
| Ct-MDR1a | CGGTTGTTTTGTTGATTGAT | real-time PCR for Ct*MDR1* |
| Ct-MDR1b | TTCAACCAAAGTAAATCCTC |  |
| Cp-erg11F | CGAGATAATCATCAACGAACATTC | Cp*ERG11* /PCR and sequencing |
| Cp-erg11R | AAAGACCGCATTGACTACCGAT |  |
| Cp-ACT1a | CGAACGTGGTTACGGTTTCTCCACTA | Internal control of Cp real-time PCR |
| Cp-ACT1b | ACTTGACCATCTGGCAATTCGTAT |  |
| Cp-erg11a | GTACACCGTCATTACTCTACCCAACA | real-time PCR for Cp*ERG11* |
| Cp-erg11b | TGCTCCTTTCATTTACAACATCATTT |  |
| Cp-CDR1a | ATTTGCCGACATCCACCGTTAGG | real-time PCR for Cp*CDR1* |
| Cp-CDR1b | ACCATGCTGTTTGCGAGTCCA |  |
| Cp-MDR1a | GATTTTTCGCTAGTCCGTGTTTG | real-time PCR for Cp*MDR1* |
| Cp-MDR1b | TGTAGGCGCATAGGTCTCAGGT |  |
| PDR1-F | GGTAAAGTCATTCTTTAGCTACG | Cg*PDR1* /PCR and sequencing |
| PDR1-R | TACAGGCTATGCACACTGTCT |  |
| FKS1F | ATGTCTTACAATAATAACGGAC | Cg*FKS1* /PCR and sequencing |
| FKS1-W1F | TTCTCCGATTTCAGCAGTTAC | Cg*FKS1* sequencing |
| FKS1-W2F | ACTCCAATCGAAAGAGTTCGT |  |
| FKS1-W3F | AGTTTCATCCAACTTCTAGCT |  |
| FKS1-W4F | TCAACACTGTCTTTTCCGTTG |  |
| FKS1-W5F | GATCAAGATCCTGAGAAGGAA |  |
| FKS1-W6F | TCGATGCTAACCAAGACAACT |  |
| FKS1-W7F | TGCTTTGATTTTCTACAGAGG |  |
| FKS1-W8F | CCTGGTTTCCATTTGAATAAC |  |
| FKS1-W9F | CTTCTTGGATTACAGAGACTA |  |
| FKS1R | TTATTTGATTGTAGACCAGGTC | Cg*FKS1* /PCR and sequencing |
| FKS2F | ATGTCTTACGATCAAGGTGG | Cg*FKS2* /PCR and sequencing |
| FKS2-W1F | CAAGGTCAAATGCCACAACAA | Cg*FKS2* sequencing |
| FKS2-W2F | ACAAAAAAGCAATGGAAGAGG |  |
| FKS2-W3F | TCTCCTACTTTCTACACTCAC |  |
| FKS2-W4F | GATTGCTACAGATTTCATTTTG |  |
| FKS2-W5F | TGTTAAGGATACCAAGATTCTG |  |
| FKS2-W6F | TTGATGCTAACCAAGACAACTA |  |
| FKS2-W7F | CTGGTTTCCATTTGAATAACTT |  |
| FKS2-W8F | AGATGGTTATCAAGAGGTAACA |  |
| FKS2-W9F | TTGGACTCAACCAATGAGAG |  |
| FKS2R | TTATTTTATAGTGGACCAGGTCTT | Cg*FKS2* /PCR and sequencing |
